# RNases Disrupt the Adaptive Potential of Malignant Cells: Perspectives for Therapy

**DOI:** 10.3389/fphar.2019.00922

**Published:** 2019-08-16

**Authors:** Vladimir Alexandrovich Mitkevich, Irina Yu Petrushanko, Alexander Alexander Makarov

**Affiliations:** Engelhardt Institute of Molecular Biology, Russian Academy of Sciences, Moscow, Russia

**Keywords:** RNase, malignant cell, adaptation potential, external environment, redox state

## Abstract

Exogenous RNases are selectively toxic to tumor cells. The reasons for this selectivity are not quite clear and should be searched for in the properties that distinguish malignant from normal cells. During onco-transformation, cells acquire properties allowing them to adapt to the altered microenvironment, such as resistance to hypoxia, changes in intracellular pH, disruption of ion transport, reduced adhesion and increased mobility, and production of specific exosomes. These adaptation mechanisms distinguish malignant cells from normal ones and give them a competitive advantage, ensuring survival and spread in the organism. Here, we analyze if the directed cytotoxic effect of exogenous RNases is linked to the disruption of the adaptive potential of tumor cells and how it can be used in anticancer therapy.

## Introduction

Enzymes that hydrolyze RNA, ribonucleases (RNases), have several biological effects on cells. Stimulation of the growth of blood vessels, toxicity toward tumor cells, and antiviral activity are important therapeutic properties of RNases. To date, a significant number of RNases with a selective cytotoxic effect on tumor cells have been studied (Libonati et al., 2008; Makarov et al., 2008; Ardelt et al., 2009; Fang and Ng, 2011; Castro et al., 2013) making them potential therapeutic agents for cancer. However, the mechanism of their antitumor activity remains largely unclear. RNases are thought to exhibit cytotoxic activity by cleaving cellular RNA. The most accessible for them are RNAs that are not associated with proteins, i.e., transport and non-coding miRNAs. Thus, angiogenin cuts tRNA molecules inside anticodon loops, producing “stress-induced” RNA fragments, which leads to inhibition of translation initiation (Ivanov et al., 2011). The cytotoxicity of onconase, RNAse from *Rana pipiens*, is also associated with the degradation of tRNA in the cytosol of tumor cells with subsequent inhibition of protein synthesis and apoptosis (Saxena et al., 2002). Сytotoxic RNAse α-sarcin from *Aspergillus oryzae* specifically cleaves one bond in 28S rRNA, causing inhibition of translation and subsequent cell death (Olmo et al., 2001). However, numerous evidences indicate that inhibition of protein synthesis is not the only cause of apoptosis induced by RNases (Qiao et al., 2012). In particular, it has been shown that binase, RNase from *Bacillus pumilus*, directly interacts with K-RAS oncoprotein, disrupting its functioning (Ilinskaya et al., 2016). No correlation was found between the decrease in the level of RNA and the toxic effect of RNases. Thus, in the precursors of myeloid cells, binase reduced the level of RNA by 20% with 67% cell viability, and in the human kidney epithelium cells, RNA levels were halved while 85% of the cells remained viable (Ilinskaya et al., 2008). In the cells of acute myeloid leukemia Kasumi-1, which are extremely sensitive to binase, the total RNA level did not change even when the viability decreased by 95% (Mitkevich et al., 2011). Onconase caused apoptosis of mitogen-stimulated lymphocytes, without reducing the level of cellular RNA (Ardelt et al., 2003).

Another argument in favor of the fact that the degradation of available RNA is not the only cause of the cytotoxicity of RNases is that they selectively eliminate certain malignant cells. It is known that the malignancy of cells occurs as a result of a change in the processes of differentiation and control of division, which may be due to the expression of oncogenes. At the same time, several similar cancerous cell lines may respond differently to RNases (Altomare et al., 2010). It was found that cells expressing certain oncogenes, like *RAS, KIT, FLT3, AML1-ETO, E6,* and *E7*, acquire sensitivity to some bacterial RNases (Ilinskaya et al., 2002; Mitkevich et al., 2010; Mitkevich et al., 2011; Mitkevich et al., 2013; Mitkevich et al., 2017). Apparently, sensitivity to RNases occurs due to the activation and/or inhibition of certain signaling pathways and changes in the properties of onco-transformed cells. It is also obvious that the rate of cell division is not a decisive criterion for the toxic action of RNases. In accordance with our hypothesis, based on the study of the molecular mechanisms of the cytotoxic effect of exogenous RNases in recent years (Mitkevich et al., 2011; Mironova et al., 2013; Mitkevich et al., 2014; Mitkevich et al., 2015; Mitkevich et al., 2017), the reason for the selective toxicity of RNase to cancer cells should be sought in the properties of tumor cells that enable them to constantly adapt to the extracellular environment. Unlike normal cells that exist in conditions of permanent tissue homeostasis, actively proliferating malignant cells affect the composition and properties of their microenvironment and must continuously adapt to changing environmental conditions. The properties of malignant cells that ensure their survival, proliferation, and distribution include altered redox status, resistance to hypoxia, changes in the functioning of ion transport systems, increased intracellular pH, reduced adhesion, and production of specific exosomes. Despite an intensive study of the toxic properties of RNases from different organisms, information about their influence on the above properties of tumor cells is rather scarce. Here, we summarize the knowledge accumulated to date about influence of RNases on adaptive properties of malignant cells and consider the directions of future investigations for RNases application in the treatment of tumors.

## RNases and Intracellular Redox Status

Cancer cells possess a redox potential different from normal cells due to their accelerated metabolism. They demand high levels of reactive oxygen species (ROS) to maintain a high proliferation rate (Sosa et al., 2013). The thiol redox status, which depends on the ratio of reduced (GSH) and oxidized (GSSG) glutathione (Gonzalez-Dosal et al., 2011; Wang et al., 2013), is also altered in cancer cells. Under normal conditions, the GSH level in cells (1–5 mM) is 100-fold higher than GSSG. Under oxidative stress, this ratio can be reduced to 1 (Allen and Mieyal, 2012). However, many tumors show elevated levels of GSH emphasizing the link between the deregulations of GSH homeostasis and cancer (Tew and Townsend, 2011). Changes in the redox status lead to changes in the functioning of redox-sensitive proteins (kinases, transcription factors, ionic transporters, etc.) due to their redox modification (Gao and Schottker, 2017). In particular, the shift of intracellular redox conditions to the oxidized state induces protein glutathionylation that protects the thiol groups of proteins from irreversible oxidation and changes their function (Mieyal et al., 2008; Miller and Mieyal, 2015). In the case of viruses that provoke oncogenesis, a change in the redox status is necessary to “tweak” the cell to fit their needs. Thus, human papillomavirus (HPV) suppresses antioxidant systems in the cell for the functioning of redox-sensitive viral oncoproteins, E6 and E7, which bind to the tumor suppressors p53 and retinoblastoma protein, respectively, suppressing their activity, which stimulates uncontrolled proliferation and prevents apoptosis. The return of cells to normal redox status will reduce the activity of such oncoproteins. Redox active drugs, which are based on changing the cell’s redox status, have already proven their effectiveness in the treatment of certain types of cancer including promyelocytic leukemia, esophageal, ovarian, non-small cell lung, colon, and breast cancer (Wondrak, 2009; Tew and Townsend, 2011). It has been established that RNases lead to a decrease in the level of ROS in tumor cells. So, onconase caused a decrease in ROS in cells of acute lymphoblastic leukemia, Jurkat, and several fibroblast cell lines (Ardelt et al., 2007). Binase caused a decrease in ROS level in Kasumi-1 cells, B16 mouse melanoma, and Jurkat cells (Mironova et al., 2013; Mitkevich et al., 2013; Burnysheva et al., 2016). RNases, by reducing ROS in tumor cells, alter the redox modifications of key proteins (such as NF-kB, p53, etc.), which, in turn, suppress the resistance of cancer cells to apoptosis. Thus, the “return” of the redox status of cancer cells by RNases to the values characteristic of normal cells lead to a decrease in their resistance to death and uncontrolled division by normalization of the redox-sensitive cellular systems.

It should be mentioned, that one of the key player in redox regulation of cells is cytosolic RNase inhibitor (RI) (Dickson et al., 2005). It binds to and inhibits extracellular RNases. It was postulated that only RNases that evade RI can kill cells. For example, RI regulates angiogenesis through very strong binding to angiogenin, which is a cytoprotective RNase. However, in the absence of RI, the angiogenin converts into a cytotoxic molecule (Thomas et al., 2018). Unlike mammalian RNases, RI does not inhibit the ribonuclease activity of cytotoxic bacterial RNases (Sevcik et al., 2002), but the absence of direct interaction between them has not been shown. RI is very sensitive to oxidative stress and is easily inactivated, while losing its ability to inhibit exogenous RNases (Dickson et al., 2005). RI inactivation can work as mechanism to switch on the RNase-mediated degradation of cellular RNA under stress conditions (Lomax et al., 2014). On the other hand, RI protects cells from oxidative stress, and its deprivation leads to decrease of GSH level as well as increase of oxidant-induced DNA damage (Monti et al., 2007). Decrease of RI activity in tumor cells induces their proliferation, increases migration and invasion, and reduces their adhesion (Chen et al., 2011). Upregulation of RI activity, on the contrary, causes the death of tumor cells (Tang et al., 2019). One can suggest that cytotoxic RNase influence on RI status by several ways. RNase reduction of ROS level, increased in tumor cells, protects RI from inactivation; exogenous RNase in cells can upregulate RI, which will reduce oxidative stress; interaction of RI with RNase may not lead to inactivation of the latter; however, it may change the properties of the RI, including protection against oxidation. These points should be clarified in future studies.

## RNases and Hypoxia

Uncontrolled division of tumor cells changes their normal microenvironment. In particular, due to the lack of blood supply, cells at a certain stage of tumor development are in a state of hypoxia. Hypoxia serves as an additional risk factor that accelerates cell malignancy. Under hypoxia and/or altered redox status of cancer cells, the transcriptional factor hypoxia inducible factor 1 (HIF1) stabilizes in them, which makes the cells resistant to oxygen deficiency. Hypoxia is a characteristic marker for the development of tumors and has a significant impact on the course of chemotherapy, often negative (Carreau et al., 2011). It can also affect the expression level of oncogenes. In particular, during hypoxia, miR29b synthesis is suppressed, which normally suppresses the synthesis of KIT oncoprotein (Liu et al., 2010) and, consequently, the level of KIT in cells increases. Activation of KIT leads to an increase in the level of HIF1 (Zhang et al., 2011). It was shown that co-expression of AML1-ETO and HIF1 oncogenes in myelogenous leukemia cells leads to a higher rate of cell proliferation *in vitro* and to a more severe course of leukemia in mice (Gao et al., 2015). By itself, stabilization of HIF1 under conditions of chronic hypoxia is a risk factor for the spread of a tumor.

The work of endogenous RNases designed to suppress the development of neoplasia is impaired under hypoxic conditions. Nature has provided a mechanism for the destruction of cells with stabilized HIF1 by increasing the level of endogenous RNase T2 (Uccella et al., 2018). It inhibits angiogenesis and induces apoptosis of malignant cells. However, in aggressive cancers, the inhibition of cell growth by RNase T2 stops working. Reduced expression of DICER, the enzyme involved in microRNA processing, is frequently observed in cancer and is associated with poor clinical outcome in various malignancies. DICER expression is suppressed by hypoxia through an epigenetic mechanism that involves inhibition of oxygen-dependent H3K27me3 demethylases KDM6A/B and results in silencing of the DICER promoter. Subsequently, reduced miRNA processing leads to de-repression of the miR-200 target ZEB1, stimulates the epithelial to mesenchymal transition, and ultimately results in the acquisition of stem cell phenotypes in human mammary epithelial cells (Van den Beucken et al., 2014). It was found that miR-630, which is upregulated under hypoxic conditions, targets and downregulates DICER expression. In an orthotopic mouse model of ovarian cancer, delivery of miR-630 using 1,2-dioleoyl-sn-glycero-3-phosphocholine nanoliposomes resulted in increased tumor growth and metastasis and decreased DICER expression (Rupaimoole et al., 2016). Exogenous RNase, resistant to inactivation under conditions of altered redox status during hypoxia, can compensate for the loss of endogenous RNase function. Binase does not contain cysteine and methionine residues and, accordingly, is insensitive to changes of redox conditions. We tested the effect of binase on Kasumi-1 cells, and cervical cancer SiHa cells growing at different oxygen contents. A decrease in [O_2_] from 21 to 5 and 1% resulted in an almost two-fold increase in the proportion of apoptotic cells in the Kasumi-1 and SiHa cells treated by binase. The increased sensitivity of cancer cells to the effect of binase under decreased oxygen level is associated with a change in the expression of oncogenes and the activation of processes mediated by oncogenic proteins. This suggests that the response of malignant cells to RNases during tumor development may be enhanced by disrupting their adaptation to low oxygen conditions.

## RNases and pH

Disruption of oxygen supply leads to aerobic glycolysis in cancer cells (Warburg effect). The excess of protons produced during glycolysis, by the Na+/H+ exchanger is transferred to the extracellular environment. Change in pH is one of the markers of cancer cells (Webb et al., 2011). Malignant cells have a “reversed” pH gradient with a constantly elevated intracellular pH that is higher than the extracellular pH (Webb et al., 2011). The increase in intracellular pH leads to the induction of cell proliferation, increases their resistance to apoptosis, and metabolically adapts the cells to oxygen deficiency. It has been suggested that a decrease in the intracellular pH of malignant cells will lead to antitumor effects (Slingerland et al., 2013). We obtained preliminary data showing that the effect of binase (0.8 µM) on Kasumi-1 cells under 20%, 5%, 1%, and 0.2% [O_2_] leads to a decrease in the intracellular pH value by 0.2–0.5 units. Since the normal functioning of tumor cells is associated with high values of intracellular pH, the decrease of this parameter under the action of RNase should disrupt the intracellular regulation and reduce their adaptive potential.

## RNases and Adhesion

To adapt to the extracellular environment, cancer cells re-arrange their plasma membranes to sustain proliferation, avoid apoptosis, and resist anticancer drugs. This leads to changes in the cell deformability, which is important for invasiveness, membrane stiffness, and receptor function causing disruption of adhesion and intercellular signaling (Bernardes and Fialho, 2018). Membrane of normal cells is characterized by a lipid asymmetry between the inner and outer leaflets. A de-regulation of this asymmetry is often encountered in cancerous cells where phosphatidylserine is often exposed in the outer membrane resulting in a negative surface charge, leading to disruption of cell adhesion and promotion of metastasis and tumor invasion (Bernardes and Fialho, 2018). It has been shown that an increase in intracellular pH is necessary for the directed migration of malignant cells (Webb et al., 2011). During oncogenic transformation of cells, there is also a change in the structure and expression of the glycoproteins and glycolipids in the cell membrane, which form the so-called adhesive molecules (Hart et al., 1991). As a result, the membrane of tumor cells contains more acid glycoproteins and phospholipids than the membrane of normal cells. This adaptation marker is one of the reasons for the selective elimination of tumor cells by RNases. Indeed, the majority of known cytotoxic RNases are basic proteins, and cationization of RNases is considered to be an effective strategy for strengthening their antitumor properties (Mitkevich et al., 2014). The proliferation of tumor cells may be associated with disruption of endogenous RNases. It has been demonstrated that endogenous RNase L inhibits cell attachment and cell spreading (Dayal et al., 2017). Prostate cancer cells depleted of RNase L show greater migration in wound healing and trans-well migration assays in response to fibronectin and serum. Over-expression of RNase L suppressed cell migration compared to both endogenous levels and knockdown cells while activation of RNase L, which requires RNase L dimerization, which inhibited cell migration. This was attributed to the destabilization of the miR-regulated transcriptome by RNase L (Rath et al., 2015). However, it was later shown that the effect of RNase L on cell migration is mediated, in part, by protein–protein interactions and does not require enzymatic activity (Dayal et al., 2017). We have previously established that binase reduces metastasis in animals (Mironova et al., 2013). This allows suggesting that binase can reduce invasion and enhances the adhesion of tumor cells. One can expect that exogenous RNases are able to influence the adhesion and motility of tumor cells, preventing the spread of the tumor. It should be borne in mind that such effects on tumor cells may have upregulation of RI, so it is absolutely necessary to verify the role of cytotoxic RNases in regulating RI activity.

## RNases and Ion Transport

**Figure 1:**
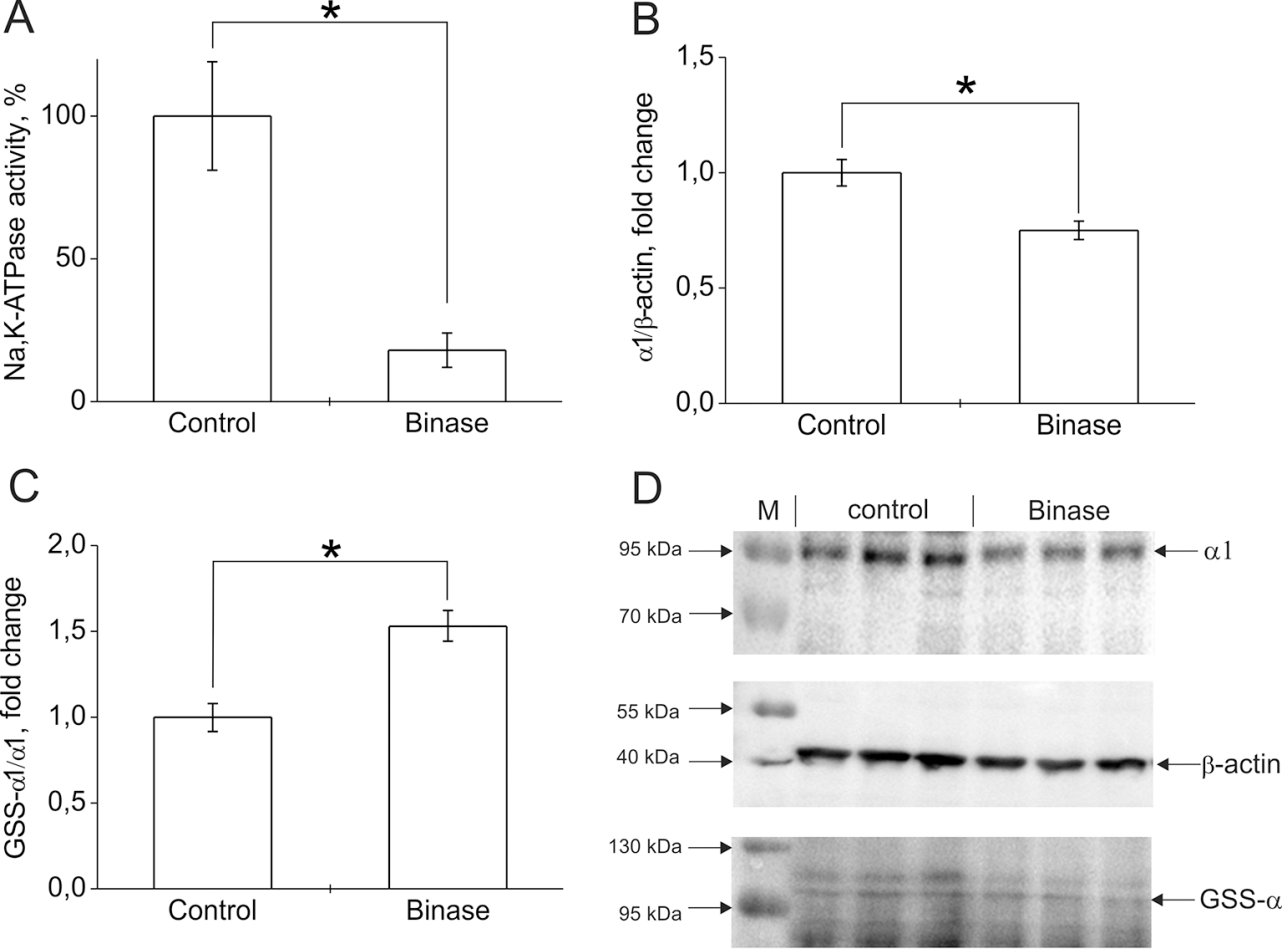
The effect of binase on Na,K-ATPase in Kasumi-1 cells after 24 h treatment of cells with 0.8 µM binase. **(A)** Effect of binase on hydrolytic activity of Na,K-ATPase. The cells after incubation with binase were destroyed by repeated freezing-thawing cycles. Hydrolytic activity of Na,K-ATPase in the cell lysates was measured as ouabain-sensitive (1 mM) ATP cleavage in the medium containing 130 mM NaCl, 20 mM KCl, 3 mM MgCl2, 3 mM ATP, and 30 mM imidazole, pH 7.4, 37°C as described in (Petrushanko et al., 2017). **(B)** The effect of binase on the amount of Na,K-ATPase in cells. Bars represent changes in the amount of Na,K-ATPase α1-subunit normalized to β-actin. **(C)** The effect of binase on the S-glutathionylation of Na,K-ATPase α1-subunit. Bars represent changes in the S-glutathionylated (GSS-α1/α1) form of the protein normalized to its total amount. The levels of S-glutathionylated Na,K-ATPase α1-subunit and total α1-subunit were estimated using immunoblotting as described in (Mitkevich et al., 2016). Mouse monoclonal anti-glutathione antibody MAB5310 (Millipore) and mouse monoclonal anti-Na,K-ATPase α1 antibody clone C464-6 (Millipore) were applied to detect glutathionylated proteins and total amount of α1-subunit correspondingly. Mouse monoclonal antibody AM4302 (Ambion) was used for β-actin detection. After staining with horseradish peroxidase–conjugated secondary antibodies, membrane was stained using a commercial kit SuperSignal West Femto Maximum Sensitivity Substrate (Thermo Scientific), and chemiluminescence was detected using Bio-Rad ChemiDoc MP instrument. Densitometric analysis was performed by Image Lab (Bio-Rad) program, and the results were represented as ratio of glutathionylated α1- subunit to total α1-subunit band intensity ([GSS- α1]/total α1) or as ratio of α1-subunit to β-actin band intensity (α1/β-actin). **(D)** The original immunoblotting readouts. Data are mean values for three independent experiments with triplicates ± standard diviation. The differences among the groups were analyzed by Student’s t-test, and p < 0.05 was considered statistically significant. Statistica 7 software was used for analysis. *p < 0.005.

Change in the functioning of ion transport systems plays a major role in the adaptability of malignant cells. The increase in intracellular pH in tumor cells is associated with changes in the activity of ionic transporters. Neoplasms induced by oncogenes or carcinogens have an abnormally high Na^+^ content, caused by membrane depolarization and the opening of potential-dependent Na^+^ channels (Lobikin et al., 2012). Membrane depolarization serves as a marker for carcinogenesis and suggests that the development of neoplastic changes can be delayed by increasing the expression of ion channels, which leads to hyperpolarization of membranes—for example, K^+^ and Cl^-^ channels. Some pharmacological antitumor agents act precisely by changing the activity of ion channels (Lobikin et al., 2012; Peigneur et al., 2012; Schweikart et al., 2013). We have found that cytotoxic bacterial RNases, binase, and 5K RNase Sa (highly cationic mutant of RNase Sa from *Streptomyces aureofaciens*) can change the functional activity of calcium-activated potassium channels artificially introduced into embryonic kidney cells (Ilinskaya et al., 2004; Ilinskaya et al., 2008). Binase leads to an increase in the level of Ca^2+^ (Mitkevich et al., 2013), which may be due to inhibition of the Na,K-ATPase, leading to the activation of the Na^+^/Ca^2+^ exchanger. Na,K-ATPase is the main ion transport system in mammalian cells. It creates a transmembrane gradient of K^+^ and Na^+^ ions in cells. This pump plays an important role in the regulation of cell volume, transmembrane potential, intracellular pH, and Ca^2+^ level through Na^+^/H^+^ and Na^+^/Ca^2+^ exchangers (Slingerland et al., 2013). In tumor cells, the activity of Na,K-ATPase is increased, which may be due to the need to remove Na^+^, which accumulates as a result of intensive activity of the Na^+^/H^+^ exchanger. In addition, maintaining the level of intracellular K^+^ with Na,K-ATPase is also necessary to suppress apoptosis. Our preliminary experiments showed that, in Kasumi-1 cells, under the action of binase (0.8 µM, 24 h), the hydrolytic activity of Na,K-ATPase was reduced by 80% (**Figure 1A**). This is due to both a 25% decrease in the level of the enzyme in cells (**Figure 1B**) and an increase in its glutathionylation level by 50% (**Figure 1C, D**). It was shown that glutathionylation led to inactivation of Na,K-ATPase and associated with a change in the cell redox status (Petrushanko et al., 2012). The decrease in the activity of Na,K-ATPase leads to a violation of ionic homeostasis and precedes the manifestation of the apoptogenic effects of binase. Obviously, this is one of the components of the mechanism of the cytotoxic effect of binase on onco-transformed cells. Thus, the effect of exogenous RNases on ion transport systems may be another reason for the selective cytotoxicity of RNases on cancer cells.

## RNases and Vesicular Transport

Exosomal vesicles secreted by tumor cell play an active role in oncogenesis and metastasis (Kim et al., 2017). They communicate between tumor cells and their microenvironment. Exosomes transporting miRNAs and proteins can cause neoplastic transformation and aid tumor development (Zhang et al., 2015). miRNAs absorbed by the recipient cell from exosomes can act as a regulator of gene expression; there is growing evidence that they are involved in the onco-transformation of cells (Kim et al., 2017). RNases bound to negatively charged molecules on the surface of cells penetrate them by endocytosis. It has been established that RNase A and BS-RNase bind actin *in vitro* and induce its polymerization (Simm et al., 1987). Since the process of polymerization/depolymerization of actin governs its dynamic properties during endocytosis, it can be assumed that RNases can influence the endocytosis of tumor cells, changing their interaction with the microenvironment.

We did not find information on how RNases influence production and composition of the exosomes of malignant cells. However, it can be assumed that since RNases change the properties of tumor cells, affecting ionic homeostasis and redox status, the number and composition of the exosomes produced by the cells will also change. An indirect confirmation of our assumptions may be the data from (Mironova et al., 2013). The authors found that RNase A reduces the level of miRNA in blood stream of mice with Luis lung carcinoma. This decrease is not related to the level of miRNA in tumor cells. MiRNA degradation by RNase A did not observed *in vitro* conditions. Given that part of extracellular miRNAs in the blood is enclosed in exosomes, it can be assumed that the decrease of miRNA level is associated with a decrease in the production of exosomes by cancer cells after treatment by RNase A. Probably, RNases affect the number and properties of RNA molecules entering into exosomes. In this case, the cancer cell will lose “communication channels” with the microenvironment, which will inevitably reduce its adaptive potential and reduce the risk of tumor spread.

## Immune-Modulatory Effects of RNases

One of the main factors in the survival of tumor cells in the organism is their ability to circumvent immune surveillance. Tumor cells may escape from immune control and proliferate in an unrestricted manner (Mittal et al., 2014). This escape can be mediated through various mechanisms, such as reduced immune recognition, increased resistance to attack by immune cells, or the development of an immunosuppressive tumor microenvironment (Mittal et al., 2014). Human extracellular RNases are mainly expressed in innate cells and display a variety of immune modulation activities (Lu et al., 2018). They can participate in host immune responses, working as alarmins and safeguard molecules against infection and inflammation. Secretion of RNase in the focus of inflammation сontributes to tissue repair and remodeling. RNases also function as cytokines and chemokines, displaying anti-inflammatory activities and inducing chemoattraction of innate cells (Lu et al., 2018). Activity of host RNases is often lowered in tumor tissue (Shlyakhovenko, 2009) and significantly reduced in serum of patients with different types of cancer (Huang et al., 2014). It turned out that exogenous RNases also have an immunomodulatory effect (Lu et al., 2018) and can be used to replenish the pool of extracellular RNases and modulate the immune response. For example, macrophage immune regulation by binase can trigger the host cell antitumor response (Makeeva et al., 2017). Binase also showed its effectiveness in restoring interferon sensitivity (IFN) of SiHa cervical cancer cells, in which the IFN response was initially suppressed by HPV (Mitkevich et al., 2017).

## Disruption of Tumor Cell Adaptability by RNases as a Potential Cancer Therapeutic Target

**Figure 2:**
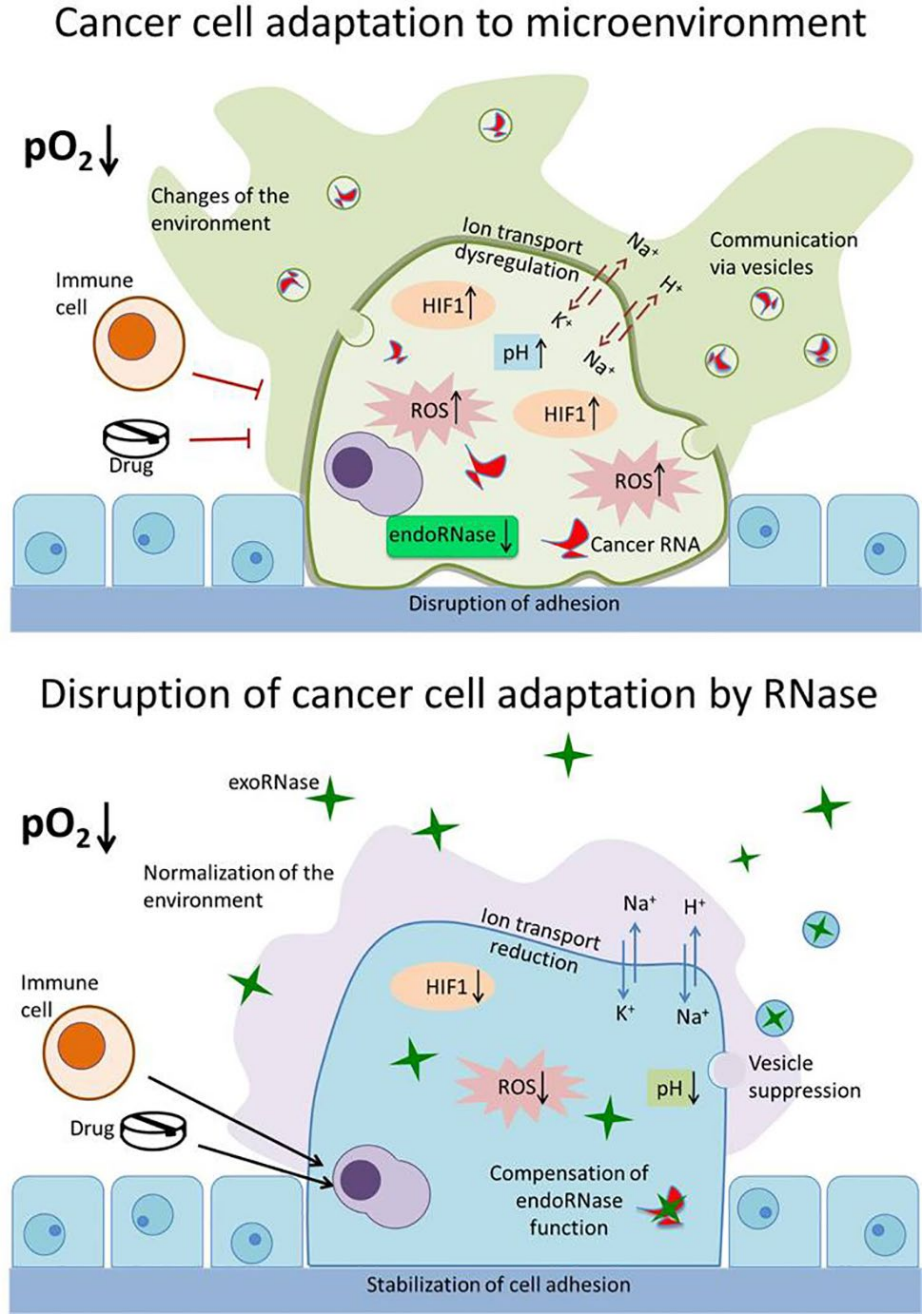
Disruption of the adaptation mechanisms of cancer cells by RNase. Upper panel: cancer cell adapts to the environment (decreased pO_2_ level) and changes it for itself, thus avoiding the immune system cells and drugs. The properties of malignant cell that ensure its survival, proliferation, and spread include altered redox status (increased reactive oxygen species [ROS]), resistance to hypoxia (hypoxia inducible factor [HIF1] increased and stabilized), changes in the functioning of ion transport systems (dysregulation of N^+^,K^+^ and H^+^,K^+^ pumps and exchangers), increased intracellular pH value, reduced adhesion, disruption of endogenous RNases (endoRNase), and production of specific exosomes (vesicles) containing specific cancer miRNA. Lower panel: exogenous RNases (exoRNase, green stars) act on a whole range of adaptive properties of tumor cells—decrease level of HIF1, ROS, and pH value; reduce activity of ion transporters; suppress exosomes production and degrade miRNA; and stabilize cell adhesion. These make tumor cells available for elimination by the immune system and reduce their drug resistance.

From the presented data, it is evident that the influence of exogenous RNases on the adaptation of malignant cells to the external environment has not been properly explored. Such adaptation mechanisms give malignant cells competitive advantages, ensuring their survival and distribution in the body. Influencing these mechanisms with the help of exogenous RNases will allow the weakening of the adaptation potential of tumor cells, inducing their death and making them more susceptible to therapeutic agents (**Figure 2**). A comprehensive study of the adaptive properties of cells that are influenced by RNases will determine the mechanisms of their selective cytotoxicity to malignant cells and establish conditions affecting the effectiveness of their antitumor activity. This will make it possible to effectively use RNases in combination with other therapeutic agents to which the tumor cells are initially resistant. Drug resistance of tumor cells is largely related to their ability to adapt to the environment. Reducing the adaptive capacity of neoplastic cells with RNases can be an effective strategy for overcoming drug resistance. A similar approach was demonstrated by us using the example of SiHa cells, the onco-transformation of which is due to HPV. Viral oncoproteins suppress the IFN response in cells, as a result of which they become resistant to interferon therapy. Binase restores IFN signaling, enhances IFN sensitivity and apoptosis in SiHa cells (Mitkevich et al., 2017). The advantage of binase and, probably, other exogenous RNases is that under conditions of low oxygen content in the environment its antineoplastic properties are increased, which accentuates the therapeutic potential of RNase for chemo-resistant tumors. Since, RNases act on a whole range of adaptive properties of tumor cells and retain their activity even in a changing micro-environment (with altered redox status, pH, oxygen, etc.), they are promising molecules for the treatment of various types of tumors. The combination of RNases with other types of anticancer drugs can help solve the problem of drug resistance of tumor cells.

## Data Availability

All datasets generated for this study are included in the manuscript and the supplementary files.

## Author Contributions

All authors listed have made substantial, direct and intellectual contribution to the work, and approved it for publication.

## Funding

The study was supported by Russian Foundation for Basic Research (Grant #17-00-00061).

## Conflict of Interest Statement

The authors declare that the research was conducted in the absence of any commercial or financial relationships that could be construed as a potential conflict of interest.
